# Late Entry into HIV Care: Estimated Impact on AIDS Mortality Rates in Brazil, 2003–2006

**DOI:** 10.1371/journal.pone.0014585

**Published:** 2011-01-25

**Authors:** Alexandre Grangeiro, Maria Mercedes Escuder, Paulo Rossi Menezes, Rosa Alencar, Euclides Ayres de Castilho

**Affiliations:** 1 Departamento de Medicina Preventiva da Faculdade de Medicina da Universidade de São Paulo, São Paulo, Brazil; 2 Instituto de Saúde, Secretaria de Estado da Saúde de São Paulo, São Paulo, Brazil; 3 Centro de Referência e Treinamento DST/Aids, Secretaria de Estado da Saúde de São Paulo, São Paulo, Brazil; University of Cape Town, South Africa

## Abstract

**Background:**

Worldwide, a high proportion of HIV-infected individuals enter into HIV care late. Here, our objective was to estimate the impact that late entry into HIV care has had on AIDS mortality rates in Brazil.

**Methodology/Principal Findings:**

We analyzed data from information systems regarding HIV-infected adults who sought treatment at public health care facilities in Brazil from 2003 to 2006. We initially estimated the prevalence of late entry into HIV care, as well as the probability of death in the first 12 months, the percentage of the risk of death attributable to late entry, and the number of avoidable deaths. We subsequently adjusted the annual AIDS mortality rate by excluding such deaths. Of the 115,369 patients evaluated, 50,358 (43.6%) had entered HIV care late, and 18,002 died in the first 12 months, representing a 16.5% probability of death in the first 12 months (95% CI: 16.3–16.7). By comparing patients who entered HIV care late with those who gained timely access, we found that the risk ratio for death was 49.5 (95% CI: 45.1–54.2). The percentage of the risk of death attributable to late entry was 95.5%, translating to 17,189 potentially avoidable deaths. Averting those deaths would have lowered the 2003–2006 AIDS mortality rate by 39.5%. Including asymptomatic patients with CD4^+^ T cell counts >200 and ≤350 cells/mm^3^ in the group who entered HIV care late increased this proportion by 1.8%.

**Conclusions/Significance:**

In Brazil, antiretroviral drugs reduced AIDS mortality by 43%. Timely entry would reduce that rate by a similar proportion, as well as resulting in a 45.2% increase in the effectiveness of the program for HIV care. The World Health Organization recommendation that asymptomatic patients with CD4^+^ T cell counts ≤350 cells/mm^3^ be treated would not have a significant impact on this scenario.

## Introduction

Late entry into HIV care is one of the principal concerns in the fight against the AIDS epidemic [1], significantly increasing the incidence of infection, the risk of death from AIDS (especially in the first year of treatment), and health care system costs, as well as reducing the effectiveness of antiretroviral drugs [2]–[11]. The problems related to late entry into HIV care are compounded by the fact that, worldwide, the proportion of individuals who have never been tested for HIV is high—over 60% in the USA [12], Spain [13], Italy [14], Brazil [15], and various African countries [1]. However, cumulative experience has shown that the interventions to increase the rates of early diagnosis of HIV infection are effective and aid in removing known barriers to HIV testing [16]–[21], allowing individuals to gain timely access to HIV care.

The criteria adopted to define late entry into HIV care have varied from study to study [20]–[27]. There is, however, a relative consensus that these criteria should express the moment at which the lack of treatment translates to significant harm to the health of HIV-infected individuals. The parameters used in order to define late entry have therefore included a combination of clinical and immunological aspects, with the aim of identifying the phases of progression of HIV infection in which therapeutic interventions are no longer effective. Such phases represent missed opportunities, as occurs when treatment is given to patients with CD4^+^ T cell counts ≤200 cells/mm^3^ [20], [24], [26], [27] or signs and symptoms of AIDS-defining illnesses [22], [23], [25]. The World Health Organization (WHO) has recently recommended that asymptomatic patients with CD4^+^ T cell counts ≤350 cells/mm^3^ be started on antiretroviral therapy [28], a recommendation that has added new elements to the discussion of late entry into HIV care. This definition translated to a considerable increase in the estimated number of HIV-infected individuals worldwide who require specific care and access to antiretroviral drugs.

Discussions regarding late entry into HIV care and the effects of late entry on the health of HIV-infected individuals would gain more depth if they considered analyses of the impact that measures to promote timely entry into HIV care have on mortality rates and on the effectiveness of programs providing access to antiretroviral drugs. In Brazil, such analyses could avail themselves of the national information systems that collect data on all individuals treated at public health care facilities [29]. These systems are affiliated with the AIDS Brazilian Program that promotes free, universal access to antiretroviral drugs and drugs to treat opportunistic infections, as well as to CD4^+^ T cell counting and viral load determination [30]. From the moment patients enter into HIV care, these systems allow the systematization of data related to immune status, clinical status, and mortality.

The purpose of the present study was to estimate by how much the AIDS mortality rate in Brazil would have been reduced had it been possible to avert the deaths that occurred in the first 12 months of treatment and were attributable to late entry into HIV care. To that end, we calculated the prevalence of late entry criteria among HIV-infected individuals who sought treatment in public health care facilities, the risk ratio (RR) of death in late entry patients compared to patients with no criteria for late entry, the percentage of the risk of death in the first 12 months attributable to late entry into HIV care, and the number of deaths that could have been avoided had these patients gained timely access to treatment. We also compared the results obtained by applying various late entry criteria.

## Methods

### Ethics statement

The study protocol was approved by the Research Ethics Committee of the São Paulo State STD and AIDS Referral and Training Center, located in the city of São Paulo, Brazil. Because patient data were collected from the Brazilian National Ministry of Health (NMH) national information systems and analyzed anonymously after the systems had been cross-referenced, informed consent was not necessary.

### Study population and information sources

The present study was undertaken as part of the evaluation of the Brazilian response to AIDS, within the context of the commitments made by the country during the United Nations General Assembly Special Session on HIV/AIDS [29].

The study population comprised adults (≥15 years of age) living with HIV who entered into HIV care at public health care facilities in Brazil between 2003 and 2006. These individuals were monitored by the national information systems in terms of the following:

- CD4^+^ T cell count and viral load (data collected via the *Sistema de Controle de Exames Laboratoriais da Rede Nacional de Contagem de Linfócitos CD4^+^/CD8^+^ e Carga Viral*—**SISCEL**, Laboratory Test Control System of the Brazilian National CD4^+^/CD8^+^ T Lymphocyte Count and Viral Load Network)
- AIDS diagnosis (data collected via the Brazilian National Epidemiological Surveillance System and compiled in the *Sistema de Informação de Agravos de Notificação*—**SINAN**, Information System on Disease Notification)
- death (data collected via the *Sistema de Informação sobre Mortalidade*—**SIM**, Mortality Information System)

The data that are fed into these systems are collected, on a compulsory basis, during clinical treatment and laboratory tests. The forms used for data collection are standardized nationwide, and the data are electronically transferred from each health care facility to the appropriate systems.

In Brazil, CD4^+^ T cell counts are performed upon entry into HIV care and are repeated periodically according to the clinical status. The laboratory staff is responsible for delivering the relevant data to the SISCEL and simultaneously issuing the test results.

Health care facilities report cases of AIDS to the SINAN when there is laboratory evidence of HIV infection, based on three criteria: at least one AIDS-defining illness or a CD4^+^ T cell count <350 cells/mm^3^; signs and symptoms of AIDS-defining illnesses or AIDS-related diseases; and a mention of HIV infection or equivalent on the death certificate. When compared with the definition adopted by the Centers for Disease Control and Prevention, the NMH definition allows the inclusion of cases of AIDS at earlier stages of infection [31].

Mortality data are extracted from death certificates [32]. The conditions and causes of death are described in a specific field and encoded in accordance with the tenth revision of the International Classification of Diseases. In order to minimize underreporting of AIDS cases, deaths in which the official cause of death was an AIDS-defining illness but HIV infection is not mentioned on the death certificate are reviewed by professional from the state or municipal epidemiological surveillance agency.

Studies evaluating the three systems have shown high coverage rates. The rates for the SIM are approximately 90% in terms of the quality of the data (the rate of ill-defined causes of death being lower than 10%) and 83% in terms of coverage (deaths registered in the SIM in relation to the 2002 demographic estimates) [32]. For the SISCEL, the coverage rate was shown to be 82.9% in 2006, considering the proportion of laboratories that had adopted the system and regularly reported information [29]. For the SINAN, the estimated coverage rate was 83.6% in 2002 and 62.3% in 2004, the parameter adopted being the retrieval of cases by means of the SISCEL and of the *Sistema de Controle Logístico de Medicamentos* (SICLOM, System for the Logistic Control of Medications) [33]. For one city in northeastern Brazil, the coverage rate was shown to be 66.9% for the 2002–2003 period [34]. A study investigating the completeness of the information registered in the SINAN reported a coverage rate of 90%, excluding data related to social variables and sexual partners [35].

In order to analyze the data, we used probabilistic record linkage, cross-referencing the information systems, a procedure that had previously been performed by the NMH in order to improve the epidemiological surveillance system for AIDS [33]. Duplicate records were identified by matching the patient date of birth, the full name of the patient, and the name of the mother of the patient. All duplicate entries were deleted.

In order to ensure that entry into HIV care had occurred between 2003 and 2006, the date of entry was cross-referenced with that of the first distribution of antiretroviral drugs (available in the SICLOM) and that of the first viral load determination (available in the SISCEL). All patients who were identified as having entered HIV care before 2003 were considered to be “under clinical follow-up at public health care facilities” and were therefore not included in the present study.

**Figure 1:**
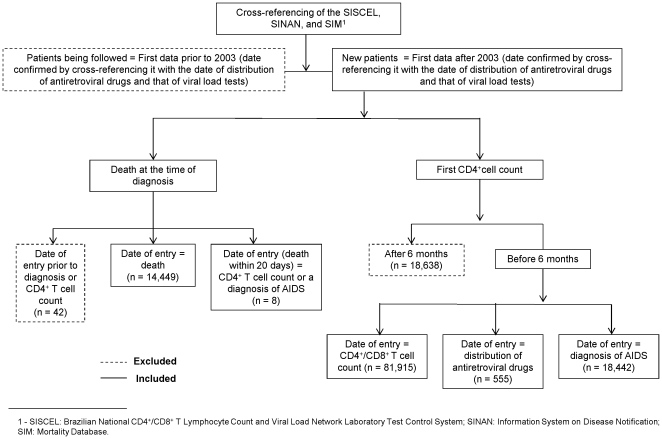
Flowchart for the inclusion of adult HIV+ in the study.

Individuals who had not undergone CD4^+^ T cell counting by the end of the sixth month of follow-up, as well as those for whom there were discrepancies between the date of death and that of entry into HIV care, were not included in the analysis. Figure 1 shows a flowchart for the inclusion of patients in the study.

### Definition of timely and late entry into HIV care

In the present study, the deaths of individuals who presented to public health care facilities too late to fully benefit from antiretroviral therapy were classified as avoidable deaths, assuming that timely initiation of antiretroviral therapy with appropriate regimens guarantees HIV-infected individuals a life expectancy close to that observed for the uninfected population [5], [36].

Patients with CD4^+^ cell counts ≤200 cells/mm^3^ or AIDS-defining illnesses at the initial examination were therefore classified as having entered into HIV care late, as were those who died soon after entry (within the first 20 days after diagnosis, which is the period of the initial hospitalization). Patients without AIDS-defining illnesses and with CD4^+^ T cell counts >200 cells/mm^3^ at the initial examination were classified as having gained timely access to HIV care.

Asymptomatic individuals with initial CD4^+^ T cell counts >200 and ≤350 cells/mm^3^ were subsequently added to the group of individuals who entered HIV care late. Those individuals were included in order to perform an analysis in which the most recent WHO recommendation for antiretroviral therapy initiation was followed [28].

The date of entry into HIV care was considered to be that on which the first data were entered into one of the information systems, assuming that the first clinical follow-up event to be registered in the information systems is the CD4^+^ T cell count. The exceptions were related to individuals who presented to public health care facilities with AIDS-defining clinical events or those who died soon after entry into HIV care. In such cases, the date of entry into HIV care was considered to be that of the diagnosis of AIDS or of death. Although uncommon, the distribution of antiretroviral drugs can occur in the first medical appointment and therefore determine the date of entry into HIV care. Figure 1 shows the number of patients included in the present study, by the source of the information regarding the date of entry into HIV care.

The first CD4^+^ T cell count was defined as that performed within six months (at most) after entry into HIV care, and AIDS-defining illnesses were included in the analysis only if diagnosed at the time of entry into HIV care. In our analysis of AIDS mortality, we considered only deaths occurring within the first 12 months after entry into HIV care and limited the analysis to those in which the death certificate listed AIDS as the underlying cause of death or the death was classified as AIDS-related on the epidemiological surveillance report. A study analyzing the accuracy of identifying deaths among AIDS patients by cross-referencing the SIM (6.4 million deaths, including all causes of death) and the SINAN reported that, between 2002 and 2005, the sensitivity, specificity, and positive predictive value of the strategy were, respectively, 87.6%, 99.6%, and 99.2% [37]. That study identified 17,488 deaths among AIDS patients. Of those deaths, 99% were registered in the SINAN.

### Data analysis

The analysis of the data was performed in five steps:

- We estimated the prevalence of late entry into HIV care based on the clinical and immunological profile at presentation.
- We calculated the probability of death from AIDS in the first 12 months of HIV care—for the population as a whole, for patients who gained timely access to HIV care, and for those who entered into HIV care late. We also estimated the RR of death as a consequence of late entry into HIV care.
- For the HIV-infected population, we estimated what percentage of the risk of death in the first 12 months was attributable to late entry into HIV care, as well as the number of deaths that could have been avoided had all individuals gained timely access to HIV care.
- We adjusted the AIDS mortality rates by excluding the avoidable deaths.
- We performed the aforementioned procedures again, this time considering asymptomatic individuals with CD4^+^ T cell counts >200 and ≤350 cells/mm^3^ as having entered HIV care late. We subsequently compared the results with those obtained previously.

In order to estimate the probability of death from AIDS in the first 12 months of HIV care, we used the Kaplan-Meier method. The log-rank test was used to compare the probability of death from AIDS in the first 12 months of HIV care for individuals who were diagnosed early with that observed for those who were diagnosed late. The level of significance was set at 0.05. The RR for death in the first 12 months due to late entry into HIV care was adopted as an effect measure and was estimated with a log-binomial regression model, with robust variance to estimate the 95% confidence interval (CI). We chose to use this procedure because most of the deaths occurred in the initial follow-up period. Patients who were lost to follow-up in the first 12 months were censored on the date of their last contact with care (last date registered in the information systems). Loss to follow-up was defined as the lack of any new entries into the information systems regarding CD4^+^ T cell count, viral load, diagnosis of AIDS, or the filling of a prescription for antiretroviral drugs for a period of more than six months. The analyses were performed with the STATA software, version 10 (Stata Corp., College Station, TX, USA).

The percentage of the risk of death in the first 12 months that was attributable to late entry into HIV care was calculated using the method proposed by Kelsey et al. [38], which is summarized by the following expression:
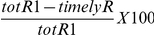
where *totR1* is the risk of death from AIDS in the first 12 months of HIV care in the HIV-infected population, and *timelyR* is the risk of death from AIDS among individuals who gained timely access to HIV care.

In order to analyze the impact that late entry into HIV care has had on the annual AIDS mortality rates, we first calculated the number of deaths that could have been avoided had all HIV-infected adults gained timely access to HIV care. To that end, we took the percentage of the risk of death that was attributable to late entry into HIV care and applied it to the total number of deaths in the first 12 months of HIV care. We then subtracted that from the total number of deaths from AIDS (nationwide) among those aged 15 years or older in the corresponding year and calculated the AIDS mortality rate adjusted by excluding the avoidable deaths. These estimates were made for each of the years in the 2003–2006 period.

## Results

Between 2003 and 2006, an annual average of 33,512 HIV-infected adults entered into HIV care via public health care facilities in Brazil, for a total of 134,049 such patients in the period as a whole. A total of 42 individuals (0.03%) were excluded because there were discrepancies related to the dates of diagnosis and death, and 18,638 (13.9%) were excluded because they had not undergone CD4^+^ T cell counting by the end of the sixth month of follow-up. Therefore, the study comprised 115,369 patients.

Among the patients analyzed, the mean and median CD4^+^ T cell counts were 378.8 cells/mm^3^ and 333.0 cells/mm^3^, respectively. Of the total of patients, 32,602 (28.3%) presented with CD4^+^ T cell counts ≤200 cells/mm^3^. Of these, 9,870 (30.3%) also had an AIDS-defining illness. In addition, 3,299 patients (2.9%) presented with CD4^+^ T cell counts >200 cells/mm^3^ and AIDS-related diseases, and 14,457 (12.5%) died soon after entry into HIV care. Therefore, late entry into HIV care was observed in 50,358 (43.6%) of the patients. Of these 50,358 patients, 34,088 (67.7%) presented with a more advanced form of the infection (more severe clinical and immunological profile), characterized by a CD4^+^ T cell count ≤100 cells/mm^3^ or death soon after entry into HIV care (Table 1).

**Table 1:** Clinical and immunological status of HIV-infected adults (≥15 years of age) upon entry into HIV care at public health care facilities in Brazil.

		2003		2004		2005		2006		2003–2006	
Clinical and immunological status		N	%	N	%	N	%	N	%	N	%
Timely		13,093	53.5	15,971	56.5	18,043	56.5	17,904	58.4	65,011	56.4
Late		11,394	46.5	12,311	43.5	13,898	43.5	12,755	41.6	50,358	43.6**
**CD4^+^ T cell count and AIDS-defining illnesses**											
**Timely**	>350 cells/mm^3^	9,511	38.8	11,798	41.7	13,354	41.8	13,091	42.7	47,754	41.4
	>200 and ≤350 cells/mm^3^ without any AIDS-defining illness	3,582	14.6	4,173	14.8	4,689	14.7	4,813	15.7	17,257	15.0
**Late**	>200 cells/mm^3^ with AIDS-defining illness	777	3.2	938	3.3	919	2.9	665	2.2	3,299	2.9
	>100 and ≤200 cells/mm^3^	2,717	11.1	3,134	11.1	3,563	11.2	3,557	11.6	12,971	11.2
	≤100 cells/mm^3^	3,740	15.3	4,507	15.9	5,961	18.7	5,423	17.7	19,631	17.0
	Death soon after entry into HIV care*	4,160	17.0	3,732	13.2	3,455	10.8	3,110	10.1	14,457	12.5
**Total**		**24,487**	**100**	**28,282**	**100**	**31,941**	**100**	**30,659**	**100**	**115,369**	**100**

*within the first 20 days after entry.

**p<0.001 for the temporal trend of the prevalence of late entry (chi-square test).

When individual years were analyzed separately, the prevalence of late entry into HIV care presented a slight reduction over the course of the period studied (p<0.001), as demonstrated by a negative variation in the overall prevalence (−4.9%), which fell from 46.5% in 2003 to 41.6% in 2006, and by a 2.4% decrease in the proportion of patients who presented to health care facilities with a more severe clinical and immunological profile (from 69.3% in 2003 to 66.9% in 2006).

Loss to follow-up in the first 12 months was observed in 16,867 patients (14.6%), 14,385 (85.3%) being lost to follow-up within the first six months of treatment. The group of patients lost to follow-up was characterized by better clinical and immunological status, as evidenced by a lower prevalence of clinical manifestations (5.8% vs. 15.0%) and a higher mean CD4^+^ T cell count (1.2 times higher). Of the individuals who gained timely access to HIV care, 12,115 (18.6%) were lost to follow-up, compared with 4,752 (9.4%) of those who entered HIV care late.

**Figure 2:**
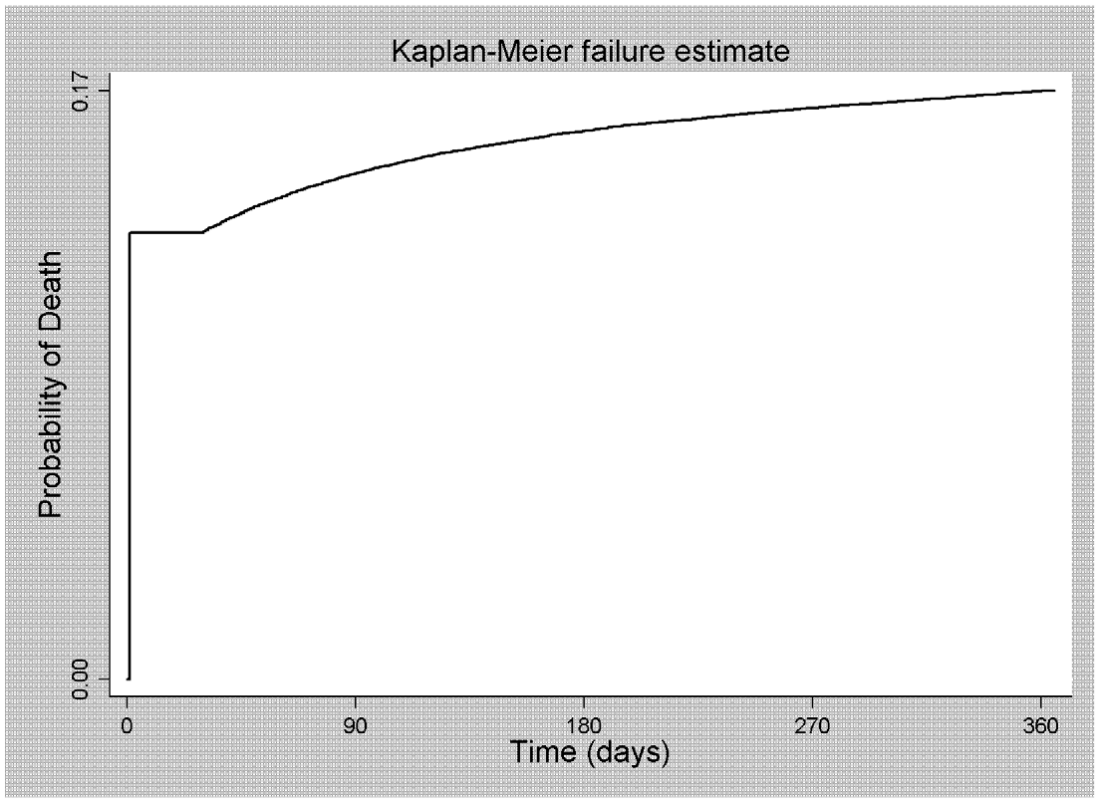
Probability of death from AIDS in the first 12 months after entry into HIV care.

A total of 18,002 deaths (15.6% of the total of patients) were recorded in the first 12 months of HIV care, translating to a 16.5% probability of death from AIDS in the first 12 months of HIV care (95% CI: 16.3–16.7; Figure 2). These deaths occurred principally in the first six months (96.3%) and presented a significant linear decrease that was inversely proportional to the duration of treatment. The proportion of individuals who died soon after their entry into HIV care (80.3% in the period under study) contributed to the fact that the deaths were concentrated in the first six months. Even after the individuals who died soon after their entry into HIV care had been excluded from the analysis, the proportional mortality in the first six months of HIV care remained at 81.2%.

**Figure 3:**
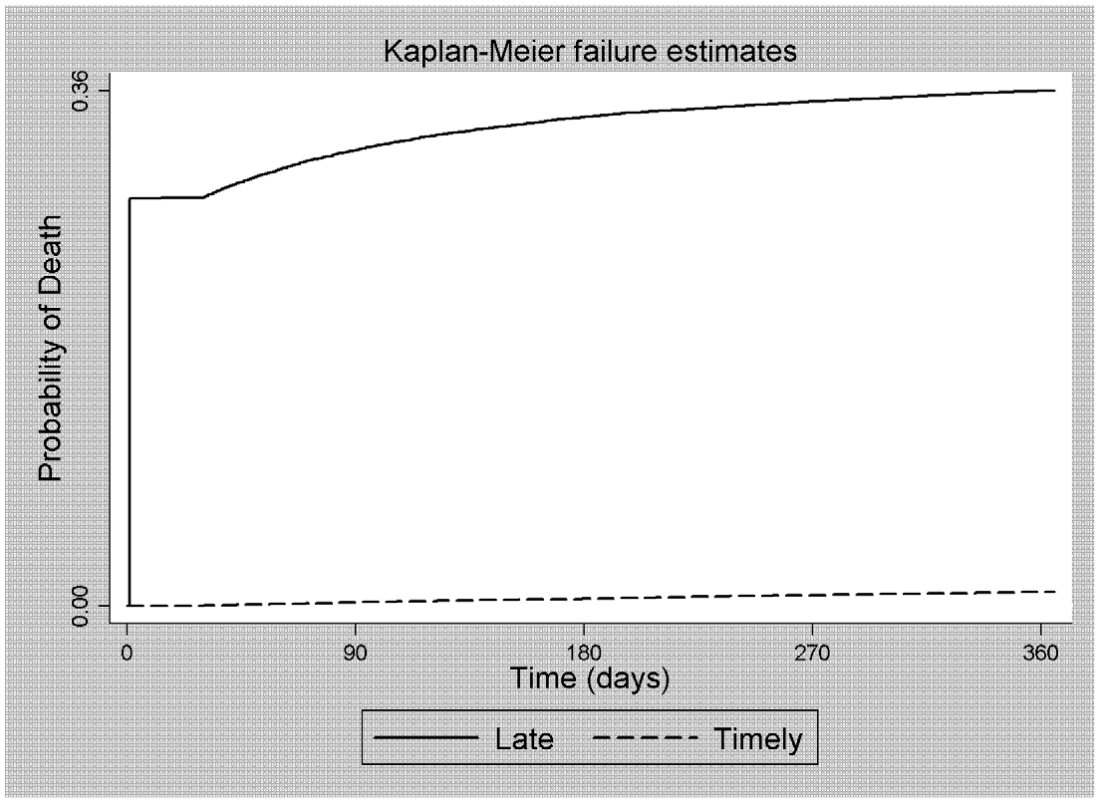
Probability of death from AIDS in 12 months, according the timing of entry into care.

Nearly all of the deaths (97.5%) occurred among the individuals who entered into HIV care late. As can be seen in Figure 3, the probability of death in the first 12 months for these patients was 36.3% (95% CI: 35.9–36.7), compared with 1.0% (95% CI: 0.9–1.1) for those who gained timely access to HIV care, corresponding to a RR of 49.5 (95% CI: 45.1–54.2; Table 2). It is of note that 97.9% of the deaths among patients who entered into HIV care late occurred among those who presented with a more severe clinical and immunological profile, had an initial CD4^+^ T cell count ≤100 cells/mm^3^, or died within the first 20 days. In the population of HIV-infected adults, 95.5% of the risk of death in the first 12 months was attributable to late entry into HIV care, meaning that 17,189 of the deaths occurring between 2003 and 2006 could have been avoided had the patients entered into HIV care while still in the early stages of HIV infection (Table 3). Among HIV-infected adults (aged 15 years or older) in Brazil, the estimated number of avoidable deaths corresponded to 39.5% of the total number of deaths (43,523) recorded between 2003 and 2006 (Table 3).

**Table 2:** Probability of death from AIDS in the first 12 months due to late entry into HIV care.

Period (months)	Late Entry (95% CI)		Timely Entry (95% CI)		Risk Ratio (95% CI)	
≤3	32.1	(31.7–32.5)	0.2	(0.2–0.3)	-	-
≤6	34.4	(34.0–34.9)	0.5	(0.4–0.5)	-	-
≤12	36.3	(35.9–36.7)	1.0	(0.9–1.1)	49.5	(45.1–54.2)

CI: confidence interval.

**Table 3:** Risk (%) of death and percentage of the risk of death in the first 12 months attributable to late entry into HIV care in the population of HIV-infected adults (≥15 years of age).

Period	Deaths in the first 12 months	Risk Population	Risk Late entry	Risk Timely entry	Percentage of risk attributable to late entry	Avoidable deaths	Total of deaths among HIV+ adults	Reduction in the total of deaths (%)
2003	4,965	20.3	42.7	0.8	96.2	4,778	11,022	43.3
2004	4,678	16.5	37.0	0.8	95.5	4,465	10,790	41.4
2005	4,523	14.2	31.4	0.9	93.8	4,243	10,872	39.0
2006	3,836	12.5	29.4	0.4	96.4	3,699	10,839	34.1
2003–2006	18,002	15.6	34.8	0.7	95.5	17,189	43,523	39.5

**Figure 4:**
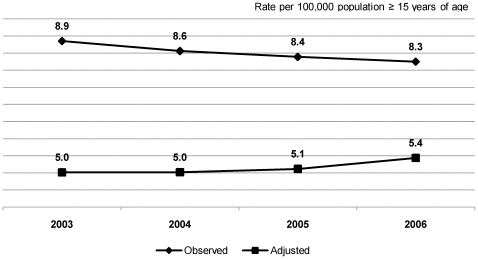
AIDS mortality rates and AIDS mortality rates adjusted by excluding avoidable deaths.

**Figure 5:**
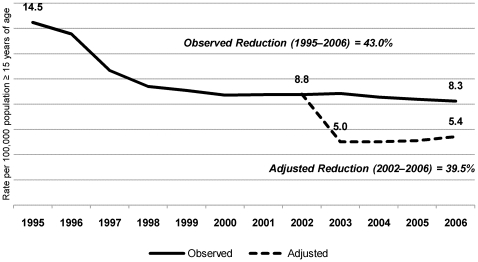
Evolution of AIDS mortality rates adjusted by excluding avoidable deaths.

For the 2003–2006 period, we adjusted the mortality rates by excluding the estimated number of avoidable deaths. We found that the hypothetical reduction ranged from 43.3% (in 2003) to 34.1% (in 2006), the mean for the period as a whole being 39.5%. In 2003 alone, that would have translated to a decrease in the number of deaths per 100,000 population (≥15 years of age) from 8.9 to 5.0 (Figure 4). Over the course of the study period, the hypothetical number of avoidable deaths trended toward a progressive decrease. In the last year of analysis (2006), the percentage of the risk of death that was attributable to late entry into HIV care was 96.4%, and the estimated number of deaths that could have been avoided had the patients gained timely access to HIV care was 3,699, equivalent to 77.4% of the total number of avoidable deaths in 2003. This reduction was associated with the annual decrease in late entry into HIV care and, consequently, with the risk of death in the first 12 months, which fell from 20.3% in 2003 to 12.5% in 2006 (Table 3). The mean proportion by which the mortality rate would have been reduced had all patients gained timely access to HIV care between 2003 and 2006 was found to be similar to the 43.0% observed between 1995, the year that preceded the introduction of antiretroviral therapy in Brazil, and 2006 (Figure 5). If, during that same period (1995–2006), all patients had also gained timely access to HIV care, the reduction in mortality would have been 62.5%, increasing the effectiveness of the HIV care program by 45.2%.

### Late entry into HIV care including asymptomatic individuals with CD4^+^ T cell counts >200 and ≤350 cells/mm^3^

The prevalence of late entry into HIV care between 2003 and 2006 increased to 58.6% when the asymptomatic individuals who presented to public health care facilities with CD4^+^ T cell counts >200 and ≤350 cells/mm^3^ (17,257; 15.0%) were included (Table 4).

**Table 4:** Comparison between two late entry criteria—inclusion and non-inclusion of asymptomatic individuals with CD4^+^ T cell counts ≤350/mm^3^—in terms of their impact on AIDS mortality rates, 2003–2006.

Indicator	Asymptomatic individuals with CD4^+^ T cell counts ≤350 cells/mm^3^ included	Asymptomatic individuals with CD4^+^ T cell counts ≤350 cells/mm^3^ not included
Probability of death within the first 12 months (timely entry)	0.4	1.0
Probability of death within the first 12 months (late entry)	26.3	36.3
Risk of death attributable to late entry	97.2	94.0
Avoidable deaths	17,497	16,963
Reduction in the total number of deaths (%)	40.2	38.9
Adjusted mortality rates (2006)	5.39	5.44

By applying the new criterion, the total number of deaths among individuals who entered HIV care late increased to 17,793 (98.8% of the total of deaths among the individuals included in the present study). The temporal distribution of the 249 deaths that occurred among asymptomatic individuals with CD4^+^ T cell counts >200 and ≤350 cells/mm^3^ was relatively homogeneous (58.2% of the deaths having occurred by the end of the sixth month). The mean CD4^+^ T cell count was 265.5 cells/mm^3^ (median, 260 cells/mm^3^; range, 203–350 cells/mm^3^) for the asymptomatic individuals who died, compared with 277.1 cells/mm^3^ (median, 270 cells/mm^3^; range, 201–350 cells/mm^3^) for those who did not.

Although the total number of deaths increased, the probability of death from AIDS in the first 12 months of HIV care, according to the new criterion, decreased to 27.6% (95% CI: 27.2–27.9) for individuals who entered HIV care late and to 0.6% (95% CI: 0.5–0.7) for those who gained timely access to HIV care. This was due to the fact that the probability of death from AIDS in the first 12 months for asymptomatic individuals with CD4^+^ T cell counts >200 and ≤350 cells/mm^3^ (1.9%; 95% CI: 1.7–2.2) was lower than was that for individuals previously classified as having entered HIV care late (RR = 0.04; 95% CI: 0.04–0.05). The RR for asymptomatic individuals with CD4^+^ T cell counts >200 and ≤350 cells/mm^3^, compared with those who gained timely access to HIV care (CD4^+^ T cell counts >350 cells/mm^3^) was 3.3 (95% CI: 2.7–3.9).

By applying the new criterion, the percentage of the risk of death in the first 12 months attributable to late entry into HIV care increased to 97.2%. This percentage corresponded to 17,497 deaths that were potentially avoidable had these individuals gained timely access to HIV care (Table 4). In addition, the percentage of avoidable deaths was 1.8% (308 deaths) higher than that observed when the former criterion was applied. As can be seen in Table 4, the mean reduction in the mortality rate increased from 39.5% (CD4^+^ T cell count ≤200 cells/mm^3^ criterion) to 40.2% (CD4^+^ T cell count ≤350 cells/mm^3^ criterion). Therefore, the mortality rates for the 2003–2006 period remained practically the same.

## Discussion

The results of the present study reveal that the risk of death from AIDS in the first 12 months was fundamentally linked to late entry into HIV care. The high proportion of individuals who entered HIV care late increased AIDS mortality rates in Brazil by more than one third. We also found that the effectiveness of the Brazilian health care program for HIV-infected individuals—considering the AIDS mortality rate as an indicator—could have increased by nearly 50% had all such individuals gained timely access to HIV care. Therefore, from a public health perspective, universal timely access to HIV care would, proportionally, have had the same impact that highly active antiretroviral drugs had when they were first introduced in the country.

The impact of late entry into HIV care on the mortality rates in Brazil was increased by the high proportion of individuals who presented at health care facilities with advanced AIDS. Approximately one third of those individuals presented with initial CD4^+^ T cell counts ≤100 cells/mm^3^ or died within the first 20 days and therefore did not benefit from the available treatments.

The relationship between late entry into HIV care and an increased risk of death (due to lower initial CD4^+^ T cell counts and a higher prevalence of AIDS-defining illnesses) has been consistently reported in various studies [6]–[10], [39]. However, in such studies, the risk of death in the first year—11% in Thailand [7], 10% in China [8], 8.4% in South Africa [9], 8.3% in Latin American countries [10], and 6.4% in low-income countries [39]—was lower than that observed in the present study. This might be attributable to the differences among the study populations (the aforementioned studies analyzed only individuals who had received antiretroviral therapy), as well as to the impact that patients who died soon after their entry into HIV care had on the mortality rates observed in the present study. It should be borne in mind that many of these patients did not receive antiretroviral therapy or received it when the probability of reconstitution of the immune response was more limited, thereby contributing to the greater risk of death. When we excluded these patients from our analysis, the risk of death in the first 12 months decreased from 16.5% (95% CI: 16.3–16.7) to 4.6% (95% CI: 4.4–4.7), a proportion that is closer to the 1.7% and 3.7% found in cohort studies of patients under antiretroviral treatment in the Brazilian cities of São Paulo [40] and Rio de Janeiro [10], respectively, as well as to the 1.8% and 6.4% found in high-income and low-income countries, respectively [39].

In general, the prevalence of late entry into HIV care in Brazil is similar to the highest rates seen in high-income countries. However, it is difficult to compare these findings due to the difference among the various studies in terms of the parameters used, which are based on different definitions of late entry. A study conducted in Barcelona showed that, between 1987 and 2006, 43.9% of patients had been tested for HIV three months before being diagnosed with AIDS [22]. A study conducted in 2005 in the USA investigated 34,424 patients and reported that 36.4% of the patients developed AIDS within the first year after being diagnosed with HIV infection [23]. In a representative sample of HIV-infected individuals diagnosed between 1996 and 2003 in France [20], 33.1% of the patients presented to health care facilities with AIDS-defining clinical events or CD4^+^ T cell counts <200 cells/mm^3^. In medium-income and low-income countries, studies of this type are scarce; however, in 17 different patient cohorts in, variously, Africa, Asia, and South America, antiretroviral treatment was started in the presence of CD4^+^ T cell counts <200 cells/mm^3^ in 77%, 78%, and 51% of the patients, respectively [24]. Between 2004 and 2005, 55% of individuals in southern Thailand presented with AIDS-related symptoms upon entry into HIV care [25].

In Brazil, two studies investigating the clientele of referral outpatient clinics, in the states of São Paulo and Minas Gerais, respectively, showed that a high proportion of patients entered into HIV care late. The study conducted in the state of São Paulo, involving 1,229 patients and carried out between 1998 and 2002, reported that 55% of the men and 38% of the women had presented with a diagnosis of AIDS [41]. In the study conducted in the state of Minas Gerais, late entry into HIV care was defined as presenting with a CD4^+^ T cell count <200 cells/mm^3^ or AIDS-defining clinical events, and the proportion of patients who received delayed antiretroviral therapy was 68.4% [26]. Another study analyzed 84,694 HIV-infected patients treated at health care facilities in Brazil and reported that 41% of such patients received delayed antiretroviral treatment, the criterion adopted for the initial prescription of antiretroviral drugs having been a CD4^+^ T cell count <200 cells/mm^3^ or symptoms of AIDS [27].

In the present study, the inclusion of asymptomatic individuals with CD4^+^ T cell counts >200 and ≤350 cells/mm^3^ increased the prevalence of late entry into HIV care by approximately 35%. However, this resulted in a less than 2% increase in the proportional reduction in the AIDS mortality rates due to the promotion of timely entry into HIV care. This minimal increase resulted from the low risk of death in the first 12 months (1.9%) observed among asymptomatic individuals with CD4^+^ T cell counts >200 and ≤ and 350 cells/mm^3^ and, in part, from the Brazilian guidelines, which, since the early 2000s, have recommended that, in specific clinical and immunological situations, antiretroviral therapy be started in patients with CD4^+^ T cell counts ≤350 cells/mm^3^ [42]. Of the asymptomatic patients with CD4^+^ T cell counts >200 and ≤350 cells/mm^3^ included in the present study, 68% used antiretroviral drugs during the study period. Among those patients, the proportional mortality in the first 12 months was approximately 4 times lower than was that observed for individuals who did not receive antiretroviral therapy (0.7% vs. 3.0%).

It should be highlighted that, even if we considered a scenario in which none of the asymptomatic patients with CD4^+^ T cell counts ≤350 cells/mm^3^ had received antiretroviral therapy during the study period and therefore assumed that the proportional mortality for the entire group was the same as that observed for patients who did not receive antiretroviral therapy, the impact of such patients on the increase in the number of avoidable deaths and, consequently, on the reduction in the mortality rates would not have been significantly greater than was that observed by adopting a CD4^+^ T cell count ≤200 cells/mm^3^ and being a symptomatic patient as late entry criteria. In that case, another 569 deaths (3.3%) would have been averted, and the proportional reduction in mortality would have increased from 39.5% to 40.8%.

The abovementioned findings bring some new elements to the discussion about the potential benefits of the WHO recommendation on AIDS mortality [28]. The studies on which the WHO based its decision used relative risks as measures of the association between early treatment and mortality. Those studies showed that the risk of death and of progression of the infection in patients who received early antiretroviral therapy was only 25% of that observed in patients who did not receive the drugs in a timely manner [28], [43]–[45]. However, the findings of the present study suggest that, from a population perspective, such benefits are not as significant, since deaths among asymptomatic patients with CD4^+^ T cell counts ≤350 cells/mm^3^ are uncommon, regardless of whether they were receiving antiretroviral therapy at the time. This might motivate researchers to conduct other studies, with the objective of establishing clinical and immunological parameters that are more accurate in identifying patients that will indeed benefit from antiretroviral therapy upon entry into HIV care (thus avoiding the excessive use of drugs), as well as of providing the basis for a definition of priorities for the use of antiretroviral therapy in a context of limited resources, prioritizing individuals at higher risk of impaired health status.

The reduction in the prevalence of late entry into HIV care in Brazil between 2003 and 2006, albeit slight, was an encouraging finding, which might be attributable to the adoption of public policies to promote early diagnosis of the infection. Studies examining the organization of health care facilities and the Brazilian response to AIDS have shown that, beginning in 2003 (when a national program encouraging individuals to be tested for HIV was launched), the number of voluntary counseling and testing centers increased [46], as did the number of diagnoses made via the public health system [29]. In addition, population-based studies in Brazil [15] have shown that the proportion of adult individuals tested for HIV increased (by 66.3%) between 1998 and 2005.

The findings of the present study should be examined in light of its limitations, which are primarily related to the data sources used. The principal limitation is related to the possibility that, due to underreporting and problems inherent to routine databases, the information systems failed to include a proportion of HIV-infected individuals in the country. This might have been caused by data entry omissions systems, by the unavailability of CD4^+^ T cell counting at the public health care facilities upon entry into HIV care, or by the fact that a proportion of patients undergo CD4^+^ T cell counting at private laboratories, meaning that some information regarding those individuals was not included in the SISCEL. In an attempt to minimize the limitations resulting from underreporting, we cross-referenced the information systems, which allowed us to retrieve a variety of information that documented entry into HIV care at public health care facilities. In addition, we chose not to analyze data collected prior to 2003. This decision was based on the fact that the coverage rates of the information systems, especially those of the SISCEL, increased significantly as of 2003. Therefore, as mentioned in the Methods section, the coverage rates of the information systems used in the present study are absolutely acceptable. In addition, the public laboratory network for CD4^+^ T cell counting has extensive coverage in Brazil, having been responsible for a mean annual number of tests, between 2003 and 2006, of 298,600 (range 257,400–325,700), translating to 1.8 tests per patient being followed via public health care facilities (median, 1.8; interquartile range: 1.3–2.1) in 2006. Even in the northern region of Brazil, which is the least economically developed region of the country and in which access to health care facilities is the most limited, this number was 1.5 (median, 1.5).

In order to determine the possible effect of underreporting, we analyzed the characteristics of 18,638 patients who had not undergone CD4^+^ T cell counting by the sixth month of follow-up. These patients were similar to the total of patients analyzed in terms of gender and age, and the proportional mortality in the first 12 months was closer to that observed in individuals who gained timely access to HIV care (1.2%). Therefore, if we had included these 18,638 individuals in the group of patients who gained timely access to HIV care, the proportional mortality in the first 12 months would have been 12.9%, and the risk of death attributable to late entry would have been 91.9%. This would have meant that the number of avoidable deaths had been overestimated by 2.5%.

The possibility that data related to individuals at greater risk of late entry into HIV care were not included in the databases represents a potential limitation and should be taken into consideration because it might have led to an underestimation of the number of avoidable deaths and of the extrapolated rate of reduction in mortality. However, the available data suggest that this is unlikely to have occurred. Studies that have analyzed underreporting in the databases of Brazil have focused on the reporting of AIDS cases, the risk of underreporting being highest for women, for individuals with a low level of education, and for patients treated at private health care facilities [47], women reportedly representing the group with the lowest risk of late entry into HIV care [27], [41]. There are no consistent data regarding individuals with a low level of education. Taking into consideration social aspects, the study conducted in the state of Minas Gerais [26] showed that there was an association between delayed antiretroviral therapy initiation and unemployment. Patients being treated at private health care facilities account for a very small proportion of the cases of HIV infection and AIDS in Brazil and therefore were not included in the present study.

It is of note that the reduction in the prevalence of late entry into HIV care and of avoidable deaths observed in the present study might be associated with a delay in the reporting of deaths among HIV-infected individuals, especially among those who require further investigation because the diagnosis of AIDS was not listed as the underlying cause of death on the death certificate. This delay is more likely to have occurred in the last year of analysis (2006). However, we believe that it is unlikely that such a delay was sufficiently long as to alter the systematic trend toward a reduction observed in all of the studies analyzed. This might have occurred if there had been a delay in the reporting of 38.7% of the deaths that occurred in 2006. In addition, it should be borne in mind that the proportional losses to follow-up are unlikely to have significantly influenced the number of deaths observed in the period, since those data were obtained from the SIM.

Despite the limitations described here, it should be borne in mind that Brazil has a broad national health care program for HIV-infected individuals (a program that has provided universal access to antiretroviral drugs since 1991), as well as information systems that allowed the monitoring of more than 130,000 patients who entered into HIV care at public health care facilities between 2003 and 2006. These aspects provide the basis for the population estimates made in the present study, and our findings allow us to suggest that addressing the issue of late entry into HIV care will have, at this juncture, in Brazil and in other countries, an impact similar to that of the introduction of highly active antiretroviral therapy.
